# Supplementary material for: Are carbon emissions trading and green financial instruments synergistic? -Comprehensive quantitative research based on content analysis
Source: PLoS One. 2024 Mar 7;19(3):e0298601. doi: 10.1371/journal.pone.0298601 (PMC10919601; doi:10.1371/journal.pone.0298601)
Supplement: S1 Data — (ZIP) [file pone.0298601.s001.zip › Data/地级市Z_F工作报告.zip!/║╙▒▒/└╚╖╗╩╨/└┤╫╘╣1⁄2╓┌║┼í╛┬φ┐╦╩2╛▌═°í┐_2005.docx]

各位代表：

现在，我代表廊坊市人民政府向大会作工作报告，请予审议，并请市政协各位委员和其他列席人员提出意见。

2004年工作回顾

过去的一年，全市人民在省委、省政府和市委的正确领导下，紧紧围绕“进位·追赶”，继往开来，奋发图强，圆满完成了市四届人大二次会议确定的各项目标任务。

2004年，全市生产总值完成605亿元，比上年增长12.5%；全部财政收入达到46亿元，增长20.4%，按可比口径增收10亿元，增长26.5%；全社会固定资产投资完成265.5亿元，增长18.3%；实际利用外资2.2亿美元，增长16.7%；外贸出口完成4.43亿美元，增长48.5%；城镇居民人均可支配收入达到9219元，农民人均纯收入达到4338元，分别增长9.7%和7%；新增就业岗位2.6万个，城镇登记失业率1.5%，低于控制目标2.5个百分点；人口自然增长率3.48‰。

——宏观调控下积极作为保发展

2004年初，按照省委“一线两厢”的发展战略，我们在全省及全国的大坐标系中定位，提出了“进位·追赶”目标。面对国家宏观调控，我们及时调整发展思路，努力破解土地、资金难题，坚持“进位·追赶”不放松。着力拓宽融资渠道，市政府邀请省11家金融机构一把手召开经济金融恳谈会，达成了100亿元的信用合作意向，实际引进资金20多亿元。利用国家开发银行19亿元贷款，重点支持电力、交通、环保、文教卫生和民营经济发展，以此带动全市信贷投放总量达到66亿元。加快资本运营步伐，促成五丰福成在上交所上市，华日家具完成在香港创业板上市准备。着力解决土地问题，对全市3万亩存量土地进行核查、清理，盘活建设用地4600亩，争取新增批地12000亩。在国家清理整顿园区过程中，经过争取，初步保留了12个园区，数量居全省之首，为保持经济持续发展赢得了主动、争取了空间。

经过努力，2004年，我们的项目建设取得了历史性突破。全市谋划运作投资千万元以上项目1015项，年度完成投资161亿元。鑫亿隆家具城、滨松光电子、电池级钴化物、达驰电气等一批亿元以上大项目相继建成，特别是华为北方产业基地等单体投资100亿元左右项目的签订，成为廊坊经济工作的点睛之笔，标志着我们的项目工作上了一个大台阶，必将对廊坊经济格局调整和长远发展产生重大影响。

——抓“四个一批”活跃经济全局

支柱产业强势发展。完成八大支柱产业发展规划，成立信息产业、会展旅游、食品工业等行业协会，各个产业增加值都增长了1/4到一倍以上。特别是东北亚暨环渤海国际合作论坛、国际金融年会两大国际性活动永久定址廊坊；印包设备、中国渔具、体育用品、国际家具、麻醉药品、国际石油天然气管道设备、消防技术与产品和“5·18”河北省经贸洽谈会、“10·18”中国农交会等九大会展，以及1500个会议的成功举办，“中国优秀旅游城市”的正式授牌，标志着廊坊会展旅游业进入了跨越式发展阶段。

龙头企业实力增强。立邦涂料、荣盛地产、卢卡斯伟利达、华日家具、神威药业、新奥集团、美联制动、汇福粮油、梅花味精等一批龙头企业实力显著增强。廊雪面粉填补了我市国家级名牌产品的空白。

园区建设发展迅猛。固安、永清两个工业园区异军突起，为廊坊城市“大三点”布局奠定了基础。固安工业园引进亚新科、正兴车轮、顺美服装等26个项目，合同引资11.2亿元，完成投资4亿元；永清燃气工业园引进项目20个，合同引资6.8亿元，完成投资2.5亿元。四个省级开发区实际利用外资2.1亿美元、内资72.8亿元，同比分别增长31.4%和10%。

基地规模快速扩张。电子信息、钢木家具、汽摩配件、印刷包装和胶合板等14个基地实力增强。

——靠重点工程提升环境平台

硬环境建设“十项工程”进展顺利。完成投资21.9亿元，建成了廊大线、香河双安路改扩建、联通CDMA三期、胜芳22万站等35个项目，启动开工了22个项目。

软环境建设“十项工程”硕果累累。全市通过ISO9000族质量管理体系认证单位超过1000家，提前完成三年目标。选送138名处级和科级干部到清华大学、中国人民大学、天津大学等高校脱产学习，对市直1000余名科级公务员进行脱产培训，引进急需专业的域外大学毕业生和中高级职称人才3390名，引进博士生42名、硕士生224名，全市人才密度达到11.14%，比上年提高0.53个百分点。京津廊区域票据清分系统业已批准。社会保障体系日趋完善，住房公积金覆盖率和归集额大幅提高，包括养老、失业、医疗、生育、工伤五大保险，以及住房公积金和城乡最低生活保障金在内的大社保资金积累达到15.4亿元，同比增长29.4%。信用体系建设顺利启动，完成了一批企业的信息录入，开通了信用信息网络。

市区“十件实事”改善了城市环境。完成投资2.35亿元，建设了市区南出口改造、南外环改造、群安街改造、城西集中供热站、人才市场、道路绿化美化等重点工程。完成40座公厕建设、50条小街小巷改造等一批便民工程。

——在统筹兼顾中实现协调发展

乡村与城镇发展逐步协调。文明生态村建设取得阶段性成果，建成115个文明生态示范村。农民税费负担比改革前下降71.4%，人均减少88.03元，减负工作排在全省首位。农业项目投入加大，全年争取国家及省农业开发资金1.3亿元。汇福粮油、福成五丰、明慧集团、梅花味精4家企业成为国家级产业化龙头企业。通过认证的无公害农产品基地达到70个，环评认证面积达80万亩。成为全国唯一实现“村村通客车”的地级市，超额完成全年“村村通油路”工程任务。“百万农民培训工程”当年培训农民38.2万人。及早出击，内防外堵，确保全市没有发生禽流感疫情。在全市90个乡镇建起87个畜牧防疫站，畜牧防疫体系建设居全省第一。

社会与经济日趋同步。《河北省2003年社会发展水平综合评价报告》显示，我市社会发展总指数位居全省第一。在此基础上，我们进一步加强科技工作。国家科技部正式命名廊坊为全国科技进步先进城市，大厂被评为全国科技进步县，香河被列为首批全国科技工作试点县，河北省创造创新学会落户廊坊。大力发展教育事业。全年投入1.4亿元，完成永清一中、霸州一中等6所高中学校改扩建；通过市场化运作，总投资2.8亿元的廊坊十四中开工；高中阶段适龄人口毛入学率由上年的53%提高到61.16%。全市10个县(市、区)在全省率先通过“两基”三轮复查。随着廊坊市电子信息工程学校、三河市职教中心、香河县职教中心的加入，我市国家级重点中等职业学校达到6家。繁荣文化艺术和体育事业。创作推出了420多件百姓喜闻乐见、歌颂美好廊坊的文艺作品，戏剧《妈妈》、《警钟》等9件获得国家级奖项。大厂评剧团获得全国“五一劳动奖状”。市博物馆、图书馆建设进展顺利。在省第十一届运动会上，实现了金牌总数从第8到第5的历史性跨越；全省第一家全民健身中心投入使用；成功承办全国拳击锦标赛等7项全国大型赛事。不断改善卫生条件。13个公共卫生项目相继开工，8个县级疾控中心投入使用，两个县医院传染病区基础工程完工；完成20个乡镇标准卫生院改建和207个村街标准卫生室建设；成为全国无偿献血先进市；全市疾病预防控制体系和突发公共事件应急处置体系取得实质进展。

人与自然更加和谐。人口得到有效控制，计划生育率达到95.4%。城市绿化覆盖率、绿地率分别达到43%和36%，人均公共绿地面积12平方米，三项指标均超过国家园林城市标准，通过国家园林城市省初评验收。市区二级以上空气质量达到330天。继通过ISO14001环境体系认证之后，再鼓干劲，乘势而上，顺利通过ISO14000国家示范区验收，这将成为廊坊一张新的城市“名片”。目前，正朝着国家环保模范城市的目标努力。

——从改革开放中增强发展活力

扎实推进各项改革。通用公司、粮油供应公司等80家国有企业完成改制，颖丽集团、三威公司等4家企业完成二次改制，国有资本退出10.44亿元，11682名国有职工实现身份转换，国企改制任务完成居全省第一。以市场化模式组建城西供热站，新增供热面积36万平方米。积极稳妥地完成了9个市政府部门的改革。组建廊坊市行政审批服务中心。取消769项行政审批事项，累计削减率达52%，居全省首位。

不断拓宽开放层面。“5·18”经贸洽谈会和“10·18”农交会承办工作在国际化、品牌化、市场化方面迈出新步伐。项目合作与京津深度对接，全年与京津合作项目达540多项，实际引资39.9亿元，引进技术项目415项，引进人才4624名，三项均占全市总量的七成以上。全面开展城市营销，成功举办廊坊“北京周”、“韩国周”、“香港周”，开通了“新浪廊坊”网站。

加快发展民营经济。新增民营企业1774家，民营经济实现增加值365亿元，占全市GDP的59%，比上年提高3个百分点；上缴税金23.8亿元，占全部税收的64%。

——以人为本建设“和谐廊坊”

精心实施“民心工程”。就业再就业工程成效明显，共筹集再就业资金4333万元，率先在全省完成下岗职工基本生活保障向失业保险并轨。出台《廊坊市被征地农民基本养老保险暂行办法》。第一次为市本级全体参保人员体检。提倡尊老爱老之风，举办“温馨敬老人、和谐进万家”主题活动。加大农村人畜饮水工程投入，解决了268个村街、18.46万人的饮水问题。宁可牺牲经济增速，也要确保群众安全，以“壮士断腕”的决心抓安全生产，确保了安全生产形势稳定。全市各类事故和死亡人数同比分别下降16.2%和11.6%。

加强精神文明和民主法制建设。大力开展群众性精神文明创建和学习型城市建设活动，市民素质和城市文明程度有了新提高。加强政府自身建设，推行政务公开，开展行风评议，推进廉政建设。自觉接受人大、政协和群众监督，3个政府部门负责人接受市人大常委会述职评议。认真办理人大代表议案、建议和政协委员提案。坚持依法治市，政府法制建设进一步加强。“平安廊坊”建设取得成效，妥善处理东方大学城的复杂问题，矛盾纠纷排查化解机制进一步完善，社会治安防控体系得到加强，维护稳定的基层基础工作更加坚实。全市交通事故、各类治安案件数量分别下降35.77%和16.23%，成为河北唯一的全国首批科技强警示范市。制定出台了突发公共事件应急处置预案总方案和21个专项预案。深入开展多种形式的普法活动，30万名青少年接受普法教育，13个村街成为全国和省民主法制建设示范村。

统计、审计、民政、民族宗教、外事侨务、信访、物价、广播电视、新闻出版、档案、地方志、人防、气象、防震减灾等工作均取得新成绩。

2004年，是极不平凡的一年。春天，我们吹响“进位·追赶”号角，秋天，“和谐廊坊”建设享誉全国。廊坊团结奋进的政治生态、清新优美的自然环境，以及弱势群体得到关爱、人民安居乐业、政府与百姓鱼水相亲的良好局面，为世人所瞩目。“和谐廊坊”的城市品牌更加响亮，廊坊人民的自豪感空前提升。

各位代表，过去的一年，成绩来之不易，是省委、省政府和市委正确领导的结果，是全市上下戮力同心、顽强拼搏的结果。在此，我代表廊坊市人民政府，向人大代表、政协委员、各民主党派、工商联和社会各界人士以及全市人民，向驻廊中省直单位、人民解放军、武警官兵和政法干警，向关心廊坊、支持廊坊的中外客商与朋友们，表示衷心的感谢！

看到成绩的同时，我们也清醒地意识到，廊坊经济社会发展中还存在着不少困难和问题。主要是经济结构有待进一步优化，经济实力有待进一步增强，社会事业有待进一步加强，“和谐廊坊”建设水平有待进一步提高。对此，我们一定高度重视，认真加以解决。

2005年目标要求

2005年是“进位·追赶”上台阶的一年，是全面实现“十五”计划的重要一年，是继续抓基层、打基础、造环境、强后劲的关键一年。

按照省委、省政府的要求，作为“一线两厢”中心位置的城市，“小廊坊要挑大担子”，主要是紧紧抓住天时、地利、人和三者兼备的大好机遇，勇敢地挑起三副担子：一是大力发展高新技术产业，努力建成全省信息产业和高新技术产业的重要基地，为全省经济结构调整做贡献；二是发挥“京津走廊，必经之坊”的优势和作用，建设京津走廊经济隆起带，为推进全省对外开放、加快环渤海经济圈合作与发展做贡献；三是总结建设“和谐廊坊”的理论与实践，把和谐社会建设推向新高度，为构建“和谐河北”做贡献。

2005年政府工作的总体要求是：以邓小平理论和“三个代表”重要思想为指导，认真贯彻党的十六大及十六届三中、四中全会和省委对廊坊工作的指示精神，全面落实市委三届八次全会各项部署，以科学发展观为统领，围绕加快“进位·追赶”、建设“和谐廊坊”，坚持不懈地优化发展环境，凝神聚力地强化项目建设，持续深入地抓好“四个一批”，积极有为地推进结构调整，努力把各项工作做精做细，全面开创经济和社会发展新局面。

2005年的主要预期目标是：地区生产总值增长12%以上；全部财政收入增长18%以上；城镇固定资产投资增长23%以上；实际利用外资2.4亿美元左右；城镇居民人均可支配收入增长8%以上，农民人均纯收入增长5%以上；城镇新增就业岗位2万个，城镇登记失业率控制在3.5%以内。

实现上述要求和目标，需要把握“五个坚持”：

一是坚持加快“进位·追赶”、建设“和谐廊坊”。“进位·追赶”是我们的目标和旗帜，“和谐廊坊”是城市的特色及品牌。我们就是要高举旗帜，光大品牌，持续推进，把“首都圈之廊坊”叫得更响。二是坚持把抓项目作为要中之要。抓项目，特别是抓好立市立县的大项目，是增强经济实力、强化“四个一批”的主要抓手，必须一招不让、步步抢先。三是坚持把环境作为第一竞争点。环境决定项目，项目促进环境。我们一定要珍惜多年来“磨剑”、“练功”的心血和成果，坚持环境和项目两手抓，实现环境与项目双促进。四是坚持以人为本、统筹发展。着眼于“黄金发展期”和“矛盾凸显期”，既要实现经济高增速，又要防止走弯路，确保发展的全面、协调、可持续；既要效率，又要公平，让城乡百姓都得到实惠。五是坚持解放思想、卧薪尝胆、做精做细、埋头苦干。廊坊发展前有标兵、后有追兵，必须不断增强机遇意识和忧患意识，以更高的工作标准、更宽广的眼界、更大胆的创新和更扎实的作风，推动政府各项工作上水平。

2005年的重点工作

完成今年的目标任务，主要做好八方面工作。

(一)以“大项目攻坚年”为抓手，深入推进“四个一批”

2005年为“大项目攻坚年”。我们要紧紧扭住“高科技、外向型”的产业发展方向，瞄准科技含量高、社会贡献大、带动能力强的大项目，集中人力、物力、财力，打一场大项目攻坚战，以此推动“四个一批”，实现“存量调强、增量调优”。

力促一批立市立县大项目。市一级突出抓一批10亿元以上的高科技、外向型立市项目，特别是全力以赴抓好首期投资20亿元的华为北方产业基地项目，确保项目进度。各县(市、区)突出抓一批1亿元以上的立县项目，确保实质性进展。

超前抓好项目运作。抓住国家和省制定“十一五”规划、京津冀都市圈区域规划的时机，谋划一批基础设施项目和社会事业项目，争取挤进国家和省规划及重点项目的盘子。做好项目基础工作，特别是搞好城市总体规划和土地利用总体规划的修编调整，利用这次最后的机会，进一步完善城市框架和功能分区，为廊坊长远发展留足空间。搞好项目组卷，不断丰富项目储备库。最大限度争取增量土地，整合和盘活存量土地，加强集约利用，鼓励现有占地企业和项目增资，鼓励园区“腾鸟换凤”，促进结构升级。

健全项目工作机制。完善领导分包重点项目制度，建立联席会议制度。推行招商引资责任制，制定《项目建设考核奖励办法》，对各单位年初定计划，年终有考核，绩效要奖惩。特别是对引来重大项目的实行重奖。设立1000万元项目招商基金，纳入年度财政预算。建立招商“绿色通道”，加强跟踪督导，提高招商效率。

创新招商方式方法。强化“招商引资是真本事、项目落地是硬政绩”的观念，鼓励各级领导带头招商，全社会共同招商。加强专业招商队伍，有园区的县(市、区)专业招商人员要达到30人以上。各级各有关部门和各工业园区根据自身规划，有针对性地实施定点、定向、分线招商。面向江、浙、粤、闽等发达地区民营资本，大力吸引内资；瞄准世界500强及发展前景广阔的好项目，大力吸引外资。在招商目标地和重点区域，做到天天有人在、月月有团组，一盯到底、咬住不放。

通过推动“大项目攻坚年”，扩充支柱产业规模，壮大龙头企业实力，提升园区经济层次，提高基地建设水平。

支柱产业：重点发展高新技术和会展旅游两大产业集群，特别是加快信息产业发展。编制高新技术产业及信息产业发展规划，细化产业定位；完善相关扶持政策；紧紧抓住华为北方产业基地，搞好全方位服务，促其尽快投产达效，下大力引进其产业配套项目；以廊坊、燕郊两个开发区为载体，面向京津及国内外知名企业、高等院校、科研院所，加大整体引进力度。通过努力，争取信息产业“一步登天、抱大金砖”。制定旅游业发展规划，开发特色旅游品种，整合各类旅游资源，积极融入环渤海旅游圈；出台加快会展旅游业发展的实施意见，开展“会展旅游推进”活动；推进一批星级酒店等配套项目建设，争取增加两家五星酒店，星级酒店达到32家以上，解决会展旅游业“卡脖子”的问题；大力引进外地知名会展旅游企业，开展会展旅游业各项认证，发挥成本、环境比较优势，提高窗口行业服务水平，增强行业竞争力；动员各方面力量吸引京津展会，实施部门吸引展会计划，一个市直部门引进5个省部级以上会议展览；以政府推动、市场化运作方式，招揽一批有影响的大型贸展活动。全年大型展览和会议争取翻一番，国家级和区域性、国际性展览达到20—30个，域外会议达到1000个以上，旅游收入达到15亿元。

龙头企业：积极开展内引外联、兼并重组、资本运作，谋划实施一批扩张企业规模的大项目，实现“三上”。“上规模”，力争销售收入10亿元以上企业达到7家。“上股市”，分头负责，强力推动，力争两家上市，3—4家完成上市辅导。“上品牌”，争创一批省级名牌产品和国家质量免检产品，力争华日、梅花等品牌成为全国驰名商标。

园区经济：各类园区要进一步优化投资环境，提高规划水平，完善功能配套，明确和培育主导产业，建立高素质的招商队伍，全力做好项目引资。以市场机制的办法，加快基础设施建设和项目招商，为园区发展注入活力；廊坊、燕郊、香河和霸州四个省级开发区制定园区投资密度和产出效益标准，进一步调整产业结构，继续打造环境体制和产业平台，规范管理，提高水平；固安、永清两个工业园区围绕主导产业和配套聚集，加大招商力度；文安、大城、大厂工业园进一步搞好规划，健全机制，高标准起步，加快发展。

基地建设：进一步强化环境、成本、资源和市场等优势，大力引进一批同行业优势企业和知名品牌，扩大规模，优化结构，提升产业层次。有针对性地扶持本地民营企业发展配套产业。

(二)继续抓好“双十工程”，促进软硬环境升级

按照“充实、调整、完善、提高”的要求，着力在做精做细上下功夫，在重点项目上求突破。

推进硬环境建设。

谋划建设10大项44个子项目，总投资49.2亿元，当年计划投资20.7亿元。

电力设施建设：新建高楼220千伏输变电工程、翟淑二回220千伏线路工程，碾子营、新镇、灵山、刘家园、信安、东沽港等110千伏输变电工程和澎耳湾110千伏二期工程。

通讯设施建设：重点建设移动GSM11期扩容、市网通公司交换机及接入网络扩容、三河宽带建设等工程。

交通设施建设：加快郊区快速路续建工程，启动贯通南北的采留线工程，力争密涿高速公路廊坊段一期工程早日开工，积极推动104国道北京段改造工程；完成农村公路“双百”工程和“村村通油路”工程；完成市区南环路、西环路等改建工程。

水利工程建设：启动永清县城供水工程，抓好农村饮水解困工程。改造治理龙河，加快市区水系建设。

公共卫生设施建设：启动建设市医院改扩建工程，续建市第三医院、市疾病预防控制中心工程，新建、改建21个乡镇卫生院。

文化教育设施建设：加快廊坊十四中及廊坊图书馆续建工程，加快廊坊一中、香河一中、文安一中、大城一中扩建工程。

市场建设：加快香河家具、大城摩配、永清蔬菜等一批专业市场建设，完成文安芦阜庄钢材市场三期工程。市区新改扩建15个农产品市场，加快农产品进大厅、进超市步伐。

生态工程建设：启动三河燕郊污水处理厂工程，完成市区南部污水管网改造、文安县垃圾处理场二期工程，完成霸州胜芳镇、永清县及三河市南、北城区污水处理厂工程。实施村庄绿化工程，全市10%以上村庄基本实现绿化。

会展旅游基础设施建设：建设国际饭店B座和文明中华城五星级酒店，完成自然公园改造工程。

工业园区基础设施建设：启动万庄新区及安次工业区建设，完善廊坊、燕郊开发区科技孵化器功能。抓好固安汽车零部件产业园、永清燃气工业园道路及电网等配套工程建设。

深化软环境建设。

信用体系建设：整合金融、工商、税务、技术监督等部门的企业信用数据，完善企业信用信息库，力争全市8万多家中小企业基础信用数据全部入库，实现信用信息实时查询。开展部分人群的个人征信工作，研究制定《廊坊市企业信用征信和评级管理办法》、《廊坊市企业信用信息查询公布办法》等一系列规范性文件。

中介服务体系建设：以劳动就业、房地产交易、技工培训、会计、审计、资产评估、科技企业孵化器等领域为重点，逐步健全和完善中介服务体系。

生产力促进中心建设：拓展服务区域，构建以廊坊市生产力中心为核心的华北地区生产力服务体系，实现跨地域、跨行业综合服务。廊坊市生产力中心力争跨入国家重点中心行列，争取1-2家县域特色和行业生产力中心列入国家级示范中心。

标准化体系建设：力争再有100个单位通过ISO9000认证，新增30个单位通过ISO14001认证，确保ISO14001环境管理体系有效运行，确保ISO14000国家示范区争创成功。

“平安廊坊”建设：深入推进平安县城、平安乡镇、平安社区、平安市场、平安单位创建活动。建立诸警联动、协调高效的新型警务机制和高效快捷的指挥调度系统，提高快速反应能力。认真抓好“十百千”工程，消除历史隐患，依法治市，力争不出新问题。搞好经常性矛盾纠纷排查，积极、稳妥地化解矛盾、处置事端。

人才高地工程建设：制定出台“十一五”人才规划，做好人才需求的分析预测；从经济社会发展需求出发，高标准完成“双千”“双百”人才引进任务，争取引进硕士以上研究生260名，其中博士50名；抓好博士后流动站、留学人员创业园等载体建设，鼓励企业吸收留学生和博士创业团队，加大科技投入力度，提升管理服务水平，使之成为吸引高层次人才的创业基地。落实吸引人才相关政策，营造栓心留人的适宜环境。全市人才总量达到27.5万人，人才密度上升到11.6%。

公共财政体系建设：深化预算管理改革，探索编制绩效预算，打破条块分割，整合资源，集中财力办大事；完善编制、经费双向控制办法，严格控制财政供养人员；积极实施国库收付制度改革，建立以国库集中收付为主要形式的财政资金缴拨方式；坚持依法治税，规范税收征管，进一步壮大财政实力。

社会保障体系建设：进一步完善养老、失业、医疗、生育、工伤保险五大社会保障体系，新增扩面参保5万人。总结北三县(市)农村低保经验，在全市范围内施行农村最低生活保障制度，争取低保人数达到4.5万人。抓好三河新型农村合作医疗试点，搭好机制框架，逐步规范提高。构建全市住房公积金统一管理和运营保障体系，大幅度提高归集额度和资金使用率，年内新增归集额2.5亿元。大社保资金累积达到17.3亿元。

金融创新工程：创新结算服务，确保5月1日正式开通京津廊“三方联网”区域资金清算模式，实现京津廊同城票据清算。积极引进民营银行，增加资本供应规模。

市场监管服务体系建设：完善行政执法部门社会联动服务系统，加大建筑、医药、食品、农资等市场整顿和规范力度，营造公平竞争、规范有序的市场环境。

(三)提升城市发展平台，增强综合竞争力

以争创全国文明城市、园林城市、绿化城市、卫生城市、环保模范城市和科技示范城市等“六项创建”为载体，围绕城市规划、建设、管理各个环节，力求精细、精致、精美。城市化率达到39%左右，提高1.5个百分点。

完善城市规划体系。完成廊坊城市总体规划修编成果及规划报批；启动廊坊市区控制性详细规划编制，逐步建立法定图则管理体系；完善各类专项规划，完成市区路网建设及城市对外交通规划，完成市区园林绿地系统和市区灯光环境总体规划；完善万庄新区规划；做好三角地保护改造规划；着手廊坊市区水环境规划；抓好龙河滨河带规划，加快安次产业功能区和东沽港新区规划建设，启动路、水、电等基础设施，加强规划、综合执法职能，推进南区开发。聘请专家对中心城市重要地段、重要建筑群和行政中心、商业中心及次中心进行整体设计，逐步形成城市设计文本体系，明晰城市风格，打造和凸显标识性城市特色。

继续办好“十件实事”。当年投资7.2亿元，围绕就业、居住、出行、教育、医疗、绿化、环保等10大项、34个子项目，改善人居环境。主要是推进光明东道、永华东道、永兴路等25项道路工程建设；改造50条小街小巷，新建10个城市游园、10座垃圾中转站，新建改建10个市场、10座卫生公厕，启动一批城中村、旧城改造项目；建成两条亮化样板街。

进一步强化城市管理。做好土地、流动人口、城市交通管理和社区建设四件事。强化土地管理，严格实行“招、拍、挂”，对房地产项目实行法定图则拍卖，提高用地效益。出台《廊坊市流动人口管理办法》，建立流动人口管理机制，合理控制规模。合理规划公交路线，增加公交班次，改善公交服务，方便市民出行；巩固“畅通工程”效果，争取达到一等管理水平。健全和完善社区卫生、服务、文化、治安等功能，提高社区自我管理、自我服务的水平。

加强小城镇建设。出台《关于加快推进城镇化战略的实施意见》及配套专件。对信安、新镇、东沽港、九州等重点建制镇进行规划编制调整，对文明生态村进行规划。用好国家开发银行贷款，加快各县(市、区)投资千万元以上的重点基础设施建设。加强县城及重点镇管理，制定《廊坊市县城及建制镇建设管理标准》、《廊坊市县城及建制镇建设管理考核暂行办法》。

(四)推进各项社会事业，努力实现可持续发展

统一规划经济、社会、人口、资源、环境发展，在工作布局和力量安排上更多地向社会事业倾斜，不断完善协调发展机制。

加快教育发展。提高“普九”水平，加大农村义务教育投入，继续实施中小学危房改造工程，新建一批标准化中小学，三河、香河、大厂三县(市)年内通过省“两基”四轮复查。以“普九”为基础向两头延伸，一头是加强学前教育，大城、文安、永清、固安、安次五县(区)力争通过省普及学前三年教育验收，全市学前三年幼儿入园率达到85%；另一头是继续普及高中阶段教育，力争全市高中阶段毛入学率超过70%，市区和北三县实现基本普及。启动千所“园林式”学校建设工程，年内完成365所建设任务。以市十四中建设为示范，优化民办教育发展环境，推动民办教育上规模、上水平。整合全市中专学校资源，逐步建立初、中、高等相互配套的职教体系。加强沟通，搞好服务，全力支持驻廊高校发展。

推动科技进步。以创建国家科技进步示范城市为统揽，启动科技创业基地建设，启动运行廊坊和燕郊高新区创业服务中心，增强科技创业、成果转化等功能；成立科技创业投资公司，形成风险投融资机制。加大科技引进和科技资源整合力度，推动中科院廊坊高新技术产业基地和清华河北研究院科技创业、研发基地建设，用足用好驻廊高校和科研院所的科技人才与科技资源。力争全市高新技术技工贸总收入增长16%，达到180亿元；科技进步贡献率提高1个百分点，达到55%。

完善医疗卫生服务体系。深化医疗体制改革，放开医疗卫生市场。积极构建医疗服务、监督执法、疾病防控和突发公共卫生事件应急处置四大体系框架。以推进新型农村合作医疗制度和完善社区医疗服务体系为重点，解决群众看病难、费用高问题。

繁荣文化艺术和体育事业。挖掘和保护廊坊传统文化资源，发展富有时代气息和廊坊特色的先进文化，加快文化产业发展，推出一批名人、名作，打造文化品牌。加大各县(市、区)文化馆、图书室建设力度，加强农村和城市社区文化建设。加快城乡体育设施建设，建设市体育场；开展“全民体质监测年”活动，推进全民健身工程；吸引和办好一批高水平重大体育赛事，全力备战省第十二届运动会。

创新环保工作。围绕建设国家环保模范城，着力推进“蓝天、碧水、净土、绿色、宁静”五大工程。进一步加大国、省干线两侧和重点区域、重点企业污染治理。加强中心城市污水治理，完善城区西部污水管网工程，谋划实施雨污分流、雨水利用工程；推进燃气化，努力增加空气一级天数。继续推进县级污水处理厂、垃圾处理场、环境监测站“三项建设”。发展生态经济、循环经济，开展生态、健康型社区和清洁生产绿色单位创建活动。

加强人口与计划生育工作。加强人口发展战略研究，制定包括外来人口在内的人口中长期发展规划，严格控制全市总人口和中心市区人口规模，探索和确定最佳的人口区间。全市人口出生率控制在12‰以内。加强出生人口性别比治理。深入实施“计生惠民”行动，落实农村独生子女父母奖金，救助计划生育困难家庭。

继续做好统计、审计、物价、民政、民族宗教、外事侨务、老干部、信访、广播电视、新闻出版、档案、地方志、人防、气象、防震减灾等工作。抓好双拥共建和优抚安置工作。关心支持妇女、儿童、残疾人和老龄事业发展。

(五)认真解决“三农”问题，统筹城乡协调发展

立足“大农村、小城市”的基本市情，着眼区域和城乡发展全局，大力提高农业产业化、农村工业化和农村城市化水平，强化县域经济支撑。力争农业产业化经营率达到57%，提高1.5个百分点；农业科技进步贡献率达到53%，提高1个百分点。

壮大县域经济。主攻民营经济，培育特色经济，壮大园区经济。加强路网、水电等基础设施建设，强化产业配套功能，集中精力招商引资。针对各县(市、区)特点，加强具体指导，加大扶持力度，推动县域经济重新洗牌，努力形成你追我赶、生龙活虎的竞争局面。

抓好文明生态村建设。以文明生态村建设为统揽，“三个文明”一起抓，全面提高农村管理水平，年内再创建300个文明生态村。同时，抓好广阳、安次、永清、固安、霸州、文安、大城7个生态家园富民工程示范县工作。

加大农业扶持力度。认真落实各项惠农政策，发挥政策效应。进一步实施“两减免、三补贴”，农业税税率再降2个百分点，继续增加对种粮农民良种和农机具购置补贴。开展涉农收费和农资市场专项治理，稳定粮食和农业生产资料价格。市级财政预算投入4382万元，比上年增长13.5%，重点用于农田水利、优质粮食产业、农村“六小”工程等基础设施建设。加强农田保护，稳定粮食播种面积，提高粮食综合生产能力。力争全年农业开发资金投入1.76亿元。

推进农业产业化经营。以畜牧、蔬菜、林果花木三大产业为重点，发展生态农业、设施农业和观光农业，扶持肉牛、奶牛养殖小区建设，加快推进百万吨奶工程。重点发挥汇福、福成、伊利等龙头企业的作用，强化企业与农户的利益联结。力争全年重点项目投入15亿元，销售收入500万元以上的龙头企业达到77家。以龙头企业和各级供销社为依托，创建一批农民专业经济合作组织，提高农业专业化和社会化程度。

搞好支农服务。“百万农民培训工程”完成35万人次任务，用三年时间把乡村干部轮训一遍。创新农技推广体系，探索农业科研人员与农民合作创业机制。推广大华夏神农公司“户联网”语音信息服务系统，加快邮政“三农服务站”建设。

(六)推进改革开放，激发经济活力

发挥自身优势，在深化各项改革和提高开放层次上，走在全省前头。 全面推进各项改革。国企改革，全年力争8家国有工业企业完成改制。域内国有工业企业，力争基本实现国有股份退出、职工身份置换。国有资产监督管理体制改革，研究出台《廊坊市市属国有资产监督管理实施办法》及配套文件，加强对所有国有资产的统一监管，减少流失，提高运营效益。事业单位改革，着眼于优化结构，认真开展清理整顿，区分单位性质，采取撤销、合并、转制、脱钩等方法，以社会化为导向推进机构改革，逐步建立以聘用制度和岗位管理制度为主的用人机制，调整优化人员结构，减少财政供养人员。公用事业改革，开放市政公用事业，盘活公用事业经营性资产，重点推进供水总公司、污水处理厂、垃圾处理场、热力总公司、市政一公司、市政二公司、环路桥收费处等单位改制。粮食流通体制改革，在积极稳妥的前提下，把保留下来的企业按照国有控股、参股形式，改成有限责任公司，实行政企分开。

稳步推进农村信用社改革。认真搞好清产核资，明晰现有产权，积极推进增资扩股，调整股权结构，确保达到国家专项票据要求的条件，构建新的产权关系。清收不良贷款，依法打击逃废债行为。年底前，全市9个县级联社改为统一法人，实行一级法人体制，成立新的县级农村信用社，为组建农村合作银行创造条件，确保农村信用社在稳定中改革、在改革中发展。

不断提高开放水平。积极参与环渤海地区合作分工。按照营造环境、用好京津、错位发展的思路，抓住国内南资北移的机遇，吸引更多大集团、大公司。年内引进省外到位资金58亿元。壮大外贸主体队伍，力争全年新增外贸经营备案企业100家。完成外贸出口5亿美元。坚持以大活动造声势、促全局。推进“5·18”环渤海国际商务节和“10·18”中国国际农产品交易会等经贸会展，在组织形式、区域范围、运作模式、贸易展览等方面实现突破；承办好东北亚暨环渤海合作机制会议、国际金融论坛、20国集团财长及央行行长会议等重大活动。加大国际宣传力度。抓住华为项目落户廊坊、城市知名度迅速提升的契机，重点面向欧美产业集中地区和大公司、大财团，组织开展国际宣传活动，推介廊坊，吸引项目，在更广的范围和更高的层次上扩大廊坊知名度，提升廊坊城市品牌。力争年内启动建设出口加工区，做好廊坊高新区申报国家级高新区的准备工作。

(七)发展壮大民营经济，增强内生经济实力

象对待外资、外商那样，对待民营资本和民营企业家。不断发展壮大民营经济，增强内生经济活力，逐步形成创业主体铺天盖地、特色产业顶天立地、块状经济形成高地、市场建设花开遍地的新局面。力争民营经济营业收入增长16%以上，上缴税金增长14%以上。

强势推进思想解放。进一步大造声势，营造全民创业氛围。破除缩手缩脚、畏首畏尾的保守思想，鼓励能人投资兴业，激活全社会每个“细胞”，大家一起来，人人当老板。

优化创业环境。出台《支持民营经济发展的若干意见》，营造各种市场主体公开竞争的环境。法律法规未禁止的非公有资本均可进入，外资内资一律对待。建立健全中小企业创业辅导、信用评价、资金担保、法律援助、信息支持五大服务体系。鼓励各类主体创办中小企业信用担保机构，力争担保资金达到2.5亿元。

注重引导规范。围绕主导、特色产业，整合行业生产能力，引导鼓励企业强强合作、资产重组、股份合作，新发展一批公司制企业，组建一批企业集团，形成聚变效应。同时，创新企业组织形式，逐步变家族式管理为现代企业管理，推进企业机制嬗变。继续扶持百家重点民营企业，壮大龙头企业集群，培育龙头企业第二梯队，争取新增规模以上民营企业1000家以上。

加强企业人才队伍建设。最大限度开发企业家资源，搞好企业家人才梯队规划，实施千名企业家培训工程。完善用人体制，鼓励人才智力入股，重点引进和培育一批职业经理人和专业经营人才。强化企业家服务社会意识，鼓励依法纳税、守法经营，全力打造优秀企业家团队。

(八)坚持以人为本，建设“和谐廊坊”

按照“以人为本、统筹协调、促进公平、保持稳定”的思路，围绕经济基础建设、民主政治建设、文化教育建设、思想道德建设、社会公平建设、平安工程建设等“六大建设”，深入实施“民心工程”，正确处理各种利益关系，扎扎实实地、一步一个脚印地把“和谐廊坊”建设推向崭新的阶段。 促进就业再就业。按照政府搭台、市场推动，就业数量与就业质量并重、临时帮扶与长效机制结合的思路，继续抓好劳动力市场建设，完善就业服务体系。实施“劳动力转移工程”，按社会用工需求，开展定向培训，推动劳动力与企业有效对接，提高就业率。研究制定促进本市劳动力就业的具体政策。落实再就业援助措施，促进下岗人员再就业。建立农村劳动力资源库，力争五年转移50万农村劳动力，年内转移农村劳动力10万人，输出劳务10万人。

关注群众生产生活问题。重点围绕农民增收、食品药品质量监管、人畜饮水安全、住房、交通、卫生环境、教育文体设施等方面，认真解决一批关系群众切身利益的热点、难点问题。严格控制医药价格和医疗服务收费，解决群众就医难问题。加强劳动保障监察执法，督促企业按时足额发放职工工资，严禁拖欠克扣农民工工资。坚决防止农村征地、城镇拆迁、企业重组改制和破产中侵害群众利益的行为，解决好失地农民的生活问题。更多的关注弱势群体，依法保障妇女、未成年人和残疾人的合法权益。

切实维护社会稳定。继续充实和完善各类重大公共卫生、自然灾害、安全事故、社会安全等突发事件应急处理预案，形成健全的应急处置工作体系。加大消防投入，推动消防事业发展。深入落实安全生产责任制，强化安全防范和隐患治理，加强对生产过程的监督管理，开展重点领域专项整治，预防和减少重特大事故发生。加强社会治安综合治理，严厉打击刑事犯罪。完善消费维权体系。依法管理宗教事务。加强信访工作，下更大功夫抓基层、打基础。

加强精神文明建设。围绕创建全国文明城市，充分运用报刊、电视、广播、互联网等载体，落实《公民思想道德建设实施纲要》，搞好社会公德、职业道德和家庭美德教育。大力开展群众性文明社区、文明村镇、文明单位等创建活动。深入推进普法教育，提高市民法律素质。开展科普教育，倡导科学、健康、文明的生活方式。

提高政府行政能力

按照“科学执政、民主执政、依法执政”的要求，围绕加快“进位·追赶”、建设“和谐廊坊”，加强行政效能建设，不断提高行政能力。

坚持科学理政。贯彻落实科学发展观和正确政绩观，坚持把当前的事情做好，把长远的事情做对。认真履行政府职责，切实转变政府职能。以加强宏观调控为契机，在确保经济总量大幅增长的同时，在调整经济结构、转变增长方式、提高经济质量和效益上多下功夫，不断提高抓发展的能力；适应新的发展阶段的需要，在抓好经济调节和市场监管的同时，围绕改善发展和人居环境，在公共管理和社会服务上多下功夫，不断提高驾驭社会复杂局面的能力；按照“小廊坊要挑大担子”的要求，以全国先进水平为标准，以更加开拓进取的精神、更加先进科学的方法和更加饱满的激情，在做精做细上多下功夫，不断提高强化自身素质的能力。各级各部门都要找准自己的位置，做到职责清、情况明、标准高、干得好，以各级各部门各项工作的创优争先保证政府总体工作上台阶。

坚持民主执政。成立市决策咨询机构，完善公众参与、专家论证、政府决策相结合的决策机制和重要决策失误责任追究制度。对涉及经济社会发展全局和群众切身利益的重大事项，广泛征询意见，保证群众依法行使民主参与和民主监督权利。自觉接受人民代表大会及其常委会的监督，接受人民政协的民主监督，认真听取民主党派、工商联、无党派人士及人民团体意见。接受新闻舆论和社会公众监督，支持监察、审计部门依法独立履行监督职责。

坚持依法施政。全面落实依法行政纲要，严格按法定权限和程序行使权力、履行职责。推进政企、政事、政资分开，充分运用市场机制。进一步制定和完善应对突发事件、保障农民权益、促进就业和完善社会保障等方面的规范性文件。做到严格执法、公正执法和文明执法。

坚持高效行政。以构建惠民亲商的服务型政府为根本，进一步加强机关作风建设，全面实施“提速、提质”工程，提速工作过程，提高工作质量。不断深化行政审批制度改革，削减审批项目，简化办事程序，压缩办理时限，办事效率继续保持全省最高。加快电子政务建设，行政审批中心、市开发区探索建立网上办事大厅。坚持继续推行政务工作问责制，建立和推行行政效能评议考核制度，加强行政监察工作和效能监察，着力解决经济管理部门和行政执法部门办事难、环节多、效率低的问题。

坚持务实勤政。注重细节品质，于细微处下功夫，把每个环节都做精彩、做到位，让每项工作都经得起实践和历史的检验。勤于学习，创建学习型机关，继续加大公务员学习、培训和多岗位锻炼的力度，用三年时间把全市政府机关干部学历提升一个档次；勤于实践，保持锐意开拓、顽强进取的气魄和干劲，勇于和善于破解各种难题、应对各类挑战、驾驭复杂局面，在实践中提高行政能力和工作水平。坚持求真务实，说实话、办实事、求实效，力戒浮躁和推诿扯皮，把更多的时间和精力，用在调查研究和工作落实上。

坚持廉洁治政。以保持共产党员先进性教育活动为契机，认真解决在思想、组织、作风以及工作方面存在的突出问题，使人民政府始终成为人民利益的忠实维护者，使广大公务员树立正确的理想信念和高尚的道德情操，永葆人民公仆的本色。坚持“标本兼治、综合治理、惩防并举、注重预防”的方针，构建教育、制度、监督并重的惩治和预防腐败体系。从决策机制、行政审批、财政管理体制、投资体制，以及严格土地制度、加强国有资产监管、规范行政执法行为和推行政务公开等八个方面，推进改革和制度创新，从源头上预防和治理腐败。与此同时，强化对行政权力的制约和监督，推进行政权力公开透明运行，逐步做到行为规范、程序严密、运行公开、结果公正、监督有力。全面开展专项资金综合治理，堵塞各种漏洞，规范资金的管理和使用。认真落实廉政建设责任制，严格执行领导干部廉洁自律各项规定。切实纠正损害群众利益的不正之风，加大查处违法违纪案件力度，树立清明进取的政府形象。

各位代表，新形势催人奋进，新任务光荣而艰巨。廊坊具备了天时、地利、人和的腾飞条件。让我们更加紧密地团结在以胡锦涛为总书记的党中央周围，高举邓小平理论和“三个代表”重要思想伟大旗帜，进一步唱响“树正气、讲团结、求发展”的主旋律，紧紧围绕加快“进位·追赶”、建设“和谐廊坊”，抓住机遇，紧咬牙关，奋力拼搏，加快发展，把廊坊建设得更加美好！
